# The multiple flavors of GoU pairs in RNA

**DOI:** 10.1002/jmr.2782

**Published:** 2019-04-29

**Authors:** Eric Westhof, Marat Yusupov, Gulnara Yusupova

**Affiliations:** ^1^ Architecture et Réactivité de l'ARN, Institut de biologie moléculaire et cellulaire du CNRS Université de Strasbourg Strasbourg France; ^2^ Department of Integrated Structural Biology, Institute of Genetics and Molecular and Cellular Biology, INSERM, U964, CNRS, UMR7104 Université de Strasbourg Illkirch France

**Keywords:** anticodon, codon, GoU pair, miscoding, mRNA, ribosome, tautomer, tRNA

## Abstract

Wobble GU pairs (or GoU) occur frequently within double‐stranded RNA helices interspersed within the standard G═C and A─U Watson‐Crick pairs. However, other types of GoU pairs interacting on their Watson‐Crick edges have been observed. The structural and functional roles of such alternative GoU pairs are surprisingly diverse and reflect the various pairings G and U can form by exploiting all the subtleties of their electronic configurations. Here, the structural characteristics of the GoU pairs are updated following the recent crystallographic structures of functional ribosomal complexes and the development in our understanding of ribosomal translation.

## 1 INTRODUCTION

GoU pairs have been previously analyzed and reviewed (see, for example, Masquida and Westhof,^1^ Varani and McClain,^2^ and Ananth et al^3^). GoU pairs, first suggested by Crick^4^ for decoding the third codon base and then called “wobble,” are since regularly observed and predicted in RNA secondary structures, and folding programs include measured energy parameters^5^ for GoU pairs. The importance of their functionality is emphasized by the high conservation of GoU pairs in critical positions in sequence alignments or in RNA structures or complexes. In self‐splicing catalytic RNAs, GoU pairs are at the cleavage site, for example, in group I introns.^6^ In RNA complexed with proteins, GoU pairs can be determinant.^2^, ^7^, ^8^ GoU pairs are also key recognition elements for small ligands (see, for example, Burgstaller et al^9^). GoU pairs are critical for long‐range packing interactions between RNA helices in crystals^10^ and in the ribosome.^11^, ^12^ An extensive analysis of GoU pairs in ribosomal structures is presented in Mokdad et al.^12^ This quick overview gives a glimpse of the multiple structural roles played by GoU pairs in RNA folding and recognition. Here, the focus is on the various arrangements between G and U that are observed in GoU pairs.

## 2 THE USUAL GOU PAIRS

**Figure 1:**
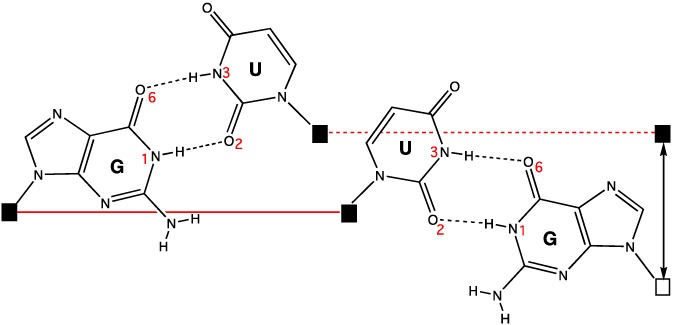
In the usual GoU wobble pair, the uracil base is displaced in the deep major groove region of a RNA double helix. In the figure, GoU and UoG pairs are compared. If one slides the C1′ atom of the G in a GoU pair on the C1′ atom of the U of a UoG pair, there is a displacement around 2 Å between the C1′ atoms of the paired base. Unlike the complementary Watson‐Crick base pairs (A─U, G═C), the GoU base pairs are not isosteric. Geometrically, isostericity between base pairs means that the positions and distances between the C1′ carbon atoms are very similar^72^

**Figure 2:**
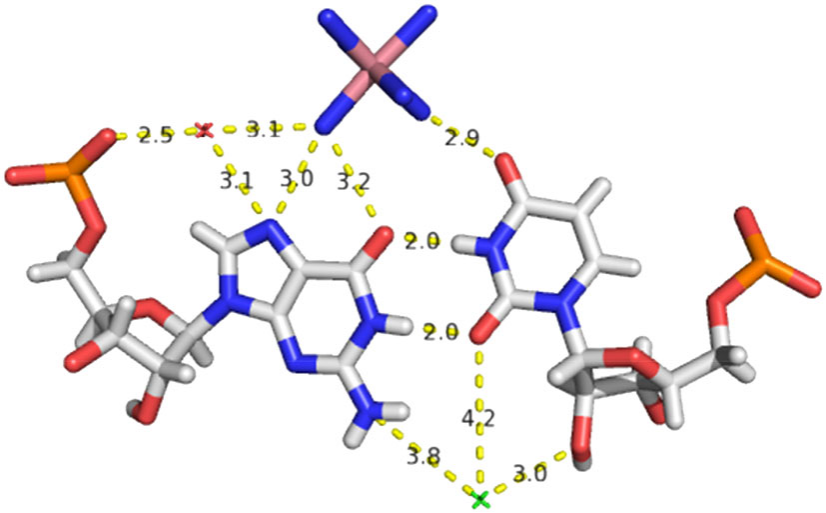
A wobble GoU pair (from PDB 4PCO^73^) with a water molecule in the minor groove (green cross) and a hydrated ion (here a cobalt hexamine) in the major groove. A water molecule linking a phosphate oxygen, the N7(G), and an ammine group is also shown. All distances are in angstrom and between the heavy atoms, except for the two base‐base H bonds

In all the examples above, the GoU pairs are canonical with the U protruding in the deep major groove of the RNA helix (Figure 1). This minor movement is at the core of the structural and functional properties of the GoU pairs. The following characteristics follow:

- GoU pairs are easily accommodated within regular RNA helices, with minimal distortions in the sugar‐phosphate backbone.
- A GoU pair is not isosteric to a UoG pair, unlike the standard G═C and A─U pairs^4^, ^13^ (Figure 1).
- The angle between the 5′GoU3′ and the following pair is undertwisted, and that between the 5′UoG3′ and the following pair is overtwisted^1^; the stacking of a 5′GoU3′ pair with the following pair in the 3′ direction is therefore more pronounced than that of a 5′UoG3′ pair.^14^
- For entering a helical stem, a 5′GoU3′ is thus more frequently observed.^3^
- The slippage of the U into the major groove leaves a cavity on the minor groove side frequently occupied by a water molecule that links the O2′(U), O2(U), and the N2(G) (Figure 2).^15^, ^16^, ^17^
- The displacement of that water molecule allows for a tighter packing with another base pair^11^ or to the insertion of a protein atom in a protein complex.^18^
- The displacement of the U also creates a binding site frequently occupied by a hydrated potassium ion with binding via hydration water molecules to O4(U), O6(G), and N7(G) in the major groove (Figure 2).^19^, ^20^

With those usual GoU pairs, it is the departure from standard Watson‐Crick pairs that is exploited for structural and functional purposes. In the complexes formed, the geometry of GoU pairs does not change conformation; at the very most, one or two water molecules exchange against a ligand in the complex. During the evolutionary processes, GoU pairs are therefore ideal transient pairs appearing between Watson‐Crick pairs in aligned sequences.^21^ Thus, in sequence alignments, conserved GoU pairs indicate a specific role either structural (stabilization) or functional as discussed above (see also Ananth et al^3^ and Mokdad et al^12^).

**Figure 3:**
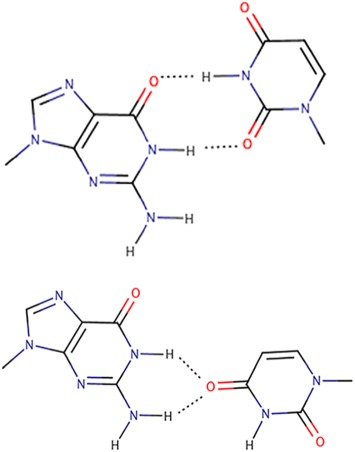
At the top, a usual GoU pair is represented and below a bifurcated GoU pair in which the O4(U) points towards the N1(G) and the N2(G) simultaneously. Such base pairs have not been found isolated and embedded within a helix, but only together with other non‐Watson‐Crick pairs. More on bifurcated base GoU pairs can be found in Leontis et al^26^

In nonhelical regions (internal or hairpin loops), GoU base pairs occur in unusual configurations called “bifurcated” where either of the uracil O4 or O2 carbonyl groups points directly to both the N1 amino and N2 amino of the guanine, for example, in the sarcin module,^22^ the UNCG tetraloop,^23^ or with a pseudouridine (Ψ) instead of U in the T‐loop of tRNAs.^24^, ^25^ The bifurcated GoU pairs (Figure 3) have not been observed yet isolated within a double‐stranded regular RNA helix in contrast to the usual GoU pairs and are found only embedded within (or framed by) other non‐Watson‐Crick pairs^26^ forming conserved loop folds or RNA modules as in the bacterial loop E of 5S rRNA.^27^ Bifurcated GoU pairs will not be discussed further in this article.

## 3 TWO ALTERNATIVE GOU PAIRS ARE OBSERVED IN RIBOSOMAL TRANSLATION

In this section, two additional alternative conformations will be described and highlighted. These alternative conformations are also accommodated within RNA helices and up to now have not been observed in other structural instances. They have been mostly observed and discussed in bacterial ribosomal ternary complexes formed between ribosome, mRNA, and tRNAs.

In the following two alternative conformations of GoU pairs, the relative dispositions of the G and U change within the functional complexes compared with the usual GoU pair. These changes are promoted upon a variation (A) in the tautomeric state of either U or G or (B) in the electronic structure of U that must be chemically modified at position C5.

**Figure 4:**
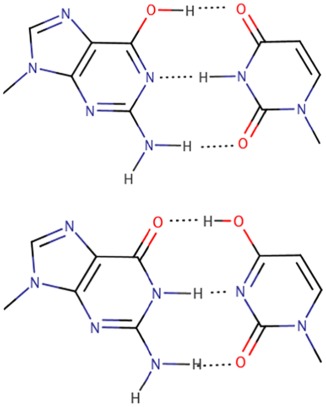
Two theoretical forms of tautomeric GoU pairs. At the top, the G adopts the enol form (and not the usual keto form), at the bottom, the U adopts the enol form (and the usual keto form). Crystallography cannot distinguish between these two possibilities. Physically, the two states are equivalent with the protons oscillating between the O6 and O4 oxygen atoms and between the N1 and N3 nitrogen atoms. Such tautomeric GoU pairs are isosteric between themselves like Watson‐Crick G═C pairs. Such tautomeric base pairs are undistinguishable from G═C pairs through interactions in the minor groove side.^30^ Such tautomeric Watson‐Crick–like pairs have been observed by crystallography with natural bases^33^, ^36^, ^38^, ^39^ or modified Uridines^52^, ^53^

- The tautomeric GoU pairs (Figure 4)

Tautomeric forms of nucleic acid bases have been discussed from the early days of structural biology^28^, ^29^ and were recently reviewed.^30^ In the past years, tautomeric GoU pairs have been implicated^31^, ^32^ but were later systematically analyzed in ternary complexes of ribosomes with mRNA and tRNAs.^33^, ^34^, ^35^, ^36^, ^37^, ^38^, ^39^ Tautomeric GoU pairs have the same shape as standard Watson‐Crick pairs, and further, they present in the minor groove exactly the same atoms as G═C pairs (Figure 4). The ribosomal decoding site has evolved for recognizing and measuring the geometry of Watson‐Crick pairs, and recognition is mediated via A‐minor interactions of two invariant As binding into the minor groove side of the first two base pairs of the triplet mini‐helix between mRNA and tRNA anticodon.^32^, ^40^ Because the ribosomal grip evolved to accommodate and bind base pairs with Watson‐Crick geometries and shapes, one can crystallize a rare event (roughly around 1 in 10 000) by presenting to the ribosome during the crystallization process only a near‐cognate tRNA.^30^, ^33^ All combinations of tautomeric GoU pairs at the first and second positions of the codon/anticodon triplets^39^ have now been crystallized within bacterial ribosomes. The types and numbers of H bonds formed between the ribosomal decoding site and GoU pairs at either the first or second position are identical to those formed between ribosomes and Watson‐Crick G═C pairs.^39^ Interestingly, miscodings because of the formation of GoU pairs between mRNA codons and near‐cognate tRNAs are the most frequently observed.^41^, ^42^, ^43^, ^44^, ^45^, ^46^, ^47^, ^48^ Tautomeric GoU pairs have also been observed using NMR techniques and their lifetimes measured in various structural environments.^49^, ^50^, ^51^

In all the preceding examples, the tautomeric GoU pairs were observed at the first or second position of coding triplets, inducing therefore a miscoding event.^39^ However, tautomeric base pairs have also been observed with modified bases at the third wobble position of codons: with a 5‐oxyacetic acid–modified U (cmo^5^U) paired to a G^52^ and with 5‐methyl‐taurine–modified U also paired to a G.^53^ In such instances, the modification of the U should facilitate or promote the adoption of a tautomeric form of the U through charge redistribution within the ring. Because of the asymmetric ribosomal grip at the third base pair of the codon/anticodon triplet, either Watson‐Crick pairs or a standard wobble GoU with the G on the anticodon (G34) and the U on the codon (U(+3)) can be accommodated. On the contrary, the reverse wobble U34oG(+3) does not accommodate well within the ribosomal grip and consequently does not translate well. However, a modified U (U*) by allowing for a tautomeric shift leads to the formation of a Watson‐Crick–like tautomeric U34*oG(+3) pair that does bind properly and is well translated.^13^, ^30^, ^54^ In summary, tautomeric GoU pairs at the first and second positions of the triple helix between codon and anticodon lead to miscoding and translational error (on average of the order of 1 in 10 000 in bacteria). However, at the third wobble position, tautomeric U*34oG(+3) pairs fit best within the tight ribosomal environment of the decoding site.

**Figure 5:**
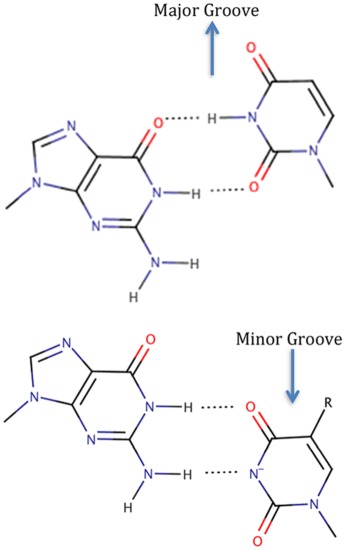
At the top is shown the usual GoU pair with the displacement of the U in the major groove, and below is shown the novel GoU pair with the U displaced into the minor groove. In the latter case, the electronic structure of the U could not be determined by X‐ray crystallography, but the shape of the base pair was clearly indicated by the electron density.^35^ In the drawing, we follow the choice made by Sochacka et al^58^ where the negative charged is shown delocalized. In the crystal structure,^35^ X = S and R = methylaminomethyl

- The minor groove–shifted GoU pairs (Figure 5)

A novel type of GoU pair was recently^36^ uncovered in crystal structure of a ternary complex between ribosome, mRNA, and a modified tRNA^Lys^. Again, that novel GoU pair occurred at the wobble position and involved a modified U34 of the tRNA anticodon with a G at the third position (G(+3)). The hypermodified U is a 5‐methylaminomethyl‐2‐thiouridine (mnm^5^s^2^U). Surprisingly, the modified U is displaced towards the minor groove (instead of the major groove as in standard wobble GoU) (see Figure 5). Importantly, this displacement renders the U34*oG(+3) pair isosteric with the G34oU(+3) pair, and consequently, the two types of base pairs occupy the same space within the ribosomal decoding site. The nature and possibility of the pairing between U34 and G(+3)‐ending codons has been a matter of discussion.^55^, ^57^ On the basis of theoretical calculations and chemical synthesis, this mode of base pairing with the modified U displaced in the minor groove was suggested.^58^ A similar model for the U34*oG(+3) pair had put been forward by Takai and Yokoyama.^56^ More recently, NMR experiments support an anionic state of the modified 5‐oxyacetic acid‐uracil base.^59^ It was therefore suggested that, like mnm^5^s^2^U34, cmo^5^U34 forms a minor groove–displaced UoG pair and not a Watson‐Crick–like tautomeric UoG pair.^52^ Further studies are required to assess which modified U*34 adopts the minor groove–shifted UoG pair or whether depending on the type of U modifications some of the pairs adopt instead a Watson‐Crick–like tautomeric geometry. Structurally, either of these two types of pairs is accommodated by the ribosomal grip and would allow for proper translation.

## 4 CONCLUSIONS

**Figure 6:**
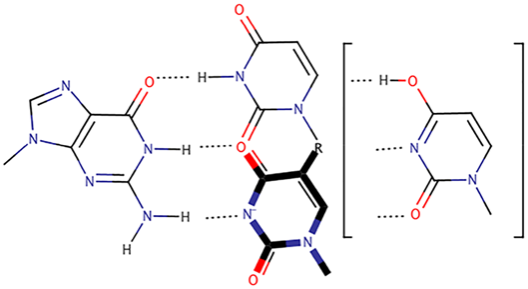
The three flavors of GoU pairs discussed: At the top, the usual mode, the GoU wobble has the U displaced into the major groove; below (thick dark lines), the anionic mode, the GoU pair has the U (that must be modified) displaced into the minor groove, and at the right, the tautomeric mode, the GoU pair is isosteric to a G═C pair

The adaptable, and almost chameleon‐like, behavior of GoU pairs is remarkable. By exploiting tautomerism, they can mimic the geometric and isosteric properties of standard complementary Watson‐Crick pairs. Indeed, they can either adopt Watson‐Crick–like pairs that appear like standard Watson‐Crick base pairs or form GoU pairs that are isosteric between them (see Figure 6). In the latter case, the U must be modified at the C5 position. This adaptable potential of GoU pairs is critically necessary for smooth and efficient ribosomal translation.^48^, ^60^, ^61^, ^62^

The nature of the chemical modification on the U base is, however, very diverse in the various organisms of the phylogeny^60^, ^63^ and reflects the historical contingencies of the evolutionary processes. Each modification at C5 of U34 (eg, cmo, mnm, or methyl‐taurine) requires the successive involvement of several modification enzymes. The cellular activities of each of those enzymes during the maturation of transfer RNAs influence the level of modification of U34 in matured functional tRNAs. Such variations allow to regulate the efficiency of ribosomal translation of particular mRNAs depending on the cellular conditions.^64^, ^65^ The evolution of the genetic code and efficient ribosomal translation is therefore intimately integrated within the biochemical, metabolic, and cellular evolution processes in extant organisms.

In this respect, it is worthwhile noting that pseudouridines (Ψ) should resist tautomeric changes and solely exist in the diketo form.^66^ Thus, a GoΨ pair is expected to occur in a single conformation, the standard wobble pair (the pair stabilizes tautomerism with altered states of both G and U, see Figure 4). Consequently, Ψ at the third position should stabilize a wobble pair (G34oΨ3), but Ψ34 could only promote translation with A(+3) (since Y34oG(+3) requires either a tautomeric form or a change in the electronic configuration of the pyrimidine). This situation occurs in eukaryotes where a second tRNA^Ile^ carrying Ψ34 decodes the (rare) AUA3 codon.^61^ Further, Ψ at the first and second positions should prevent miscoding (because tautomeric GoΨ are not expected to occur). In eukaryotes, tRNA^Tyr^ is characterized by the presence of Ψ35.^61^

The adaptable potential of GoU pairs is particularly key for a subset of codons. Indeed, for NNY codons, a G34 (modified or not) in tRNAs can decode either C(+3) or U(+3), which is not the case for NNR codons in two‐codon boxes (Arg (AGR), Gln, Glu, Leu (UUR), Lys, Trp) that require a modified U34* to decode G(+3) without impairing decoding of A(3). Evolution exploits variations in codon usage and in tRNA species for diversifying the decoding range.^54^, ^60^, ^61^, ^67^, ^68^ In order to escape from the dependence on modification enzymes acting on U34, two pathways are possible: One can either impose a strong selection against G‐ending codons or duplicate the tRNAs so that the corresponding C34‐tRNA is present for decoding G‐ending codons. The first solution is seen in fungal mitochondria^69^ and insect symbionts,^70^ and the second solution is possibly present in a sea cucumber symbiont.^71^

Such general evolutionary mechanisms have been observed in various organisms or organelles, but they could also take place within cells of multicellular organisms. Therefore, depending on the developmental stage or the cellular type, the G/C content of transcribed mRNAs could be different and, thus, the codon usage, or there could be variations in the extent of U34 modifications and the presence of other tRNA species. These general conclusions are based on the evolutionary grounded hypothesis that the key structural constraints acting at the decoding site in the ternary complexes formed between ribosome, mRNA, and tRNAs are basically similar throughout phylogeny.

## CONFLICT OF INTEREST

None.

## AUTHOR CONTRIBUTION

E. W., M. Y., and G. Y. participated in the research and wrote the paper.
